# Building bridges: simultaneous multimodal neuroimaging approaches for exploring the organization of brain networks

**DOI:** 10.1117/1.NPh.9.3.032202

**Published:** 2022-09-23

**Authors:** Evelyn M. R. Lake, Michael J. Higley

**Affiliations:** a Yale School of Medicine, Department of Radiology and Biomedical Imaging, New Haven, Connecticut, United States; b Yale School of Medicine, Departments of Neuroscience and Psychiatry, New Haven, Connecticut, United States; c Kavli Institute for Neuroscience, Yale School of Medicine, New Haven, Connecticut, United States; d Program in Cellular Neuroscience, Neurodegeneration, and Repair, New Haven, Connecticut, United States

**Keywords:** brain networks, simultaneous imaging, multiphoton imaging, single-photon imaging, fiber photometry, magnetic resonance imaging

## Abstract

Brain organization is evident across spatiotemporal scales as well as from structural and functional data. Yet, translating from micro- to macroscale (vice versa) as well as between different measures is difficult. Reconciling disparate observations from different modes is challenging because each specializes within a restricted spatiotemporal milieu, usually has bounded organ coverage, and has access to different contrasts. True intersubject biological heterogeneity, variation in experiment implementation (e.g., use of anesthesia), and true moment-to-moment variations in brain activity (maybe attributable to different brain states) also contribute to variability between studies. Ultimately, for a deeper and more actionable understanding of brain organization, an ability to translate across scales, measures, and species is needed. Simultaneous multimodal methods can contribute to bettering this understanding. We consider four modes, three optically based: multiphoton imaging, single-photon (wide-field) imaging, and fiber photometry, as well as magnetic resonance imaging. We discuss each mode as well as their pairwise combinations with regard to the definition and study of brain networks.

## 1 Introduction

The brain abides by a set of organizing principles, which can be conceptualized as interconnected circuits or networks. The features used to define brain networks can be structural or functional but must work to compartmentalize the tissue into regions that have physical or activity-based relationships. Part of the utility of brain networks is that they can be translated across subjects, populations, time, conditions, species, and modes to interrogate different scientific questions. Neuroimaging methods provide powerful strategies for monitoring neural activity at a range of spatial and temporal scales, in both human and nonhuman subjects. As each individual method has its own strengths and limitations, the combined implementation of complementary neuroimaging modes can yield access to a broader spatiotemporal axis and wider array of features. Such integrated approaches offer the means to collect simultaneous measures of spontaneous activity and generate innovative insights into neurological diseases as well as healthy brain function.^1^^–^^7^ Further, spontaneous activity constitutes the vast majority of all brain activity and is boundless in that it emerges from across the whole-brain and has been shown to provide information that is independent from stimulus-evoked activity.^8^^,^^9^

Every neuroimaging modality operates within a specific spatiotemporal milieu can access either a portion of the brain or the whole organ, and offers a limited—but specialized—arsenal of measurements. Each mode can be applied within a subset of species due to methodological requirements such as subject size or invasiveness. From modes that operate at the finest through coarsest spatiotemporal resolution, with limited or whole organ coverage, and within different species, there is evidence that the brain abides by a set of organizing principles. Gaining a deeper understanding of these principles—how they translate across species, spatiotemporal scales, and contrasts, differ between brain regions, evolve throughout the lifespan, and breakdown with injury or disease—is an overarching goal of network neuroscience.

In this review, we discuss the design and implementation of simultaneous multimodal approaches and highlight areas of brain network research where multimodal data has made a significant impact. To facilitate this discussion, we concentrate on three optical modes: multiphoton imaging, single-photon (wide-field) imaging, and fiber photometry, as well as magnetic resonance imaging (MRI). These modes cover a wide spatiotemporal range, measure diverse brain features, have variable brain coverage, and can be applied in different species. We conclude by predicting how simultaneous multimodal approaches may evolve in the near future to have a greater impact on the definition and study of brain networks. This review is intended to give a broad overview and is meant for a general scientific audience. This review is part of the Special Section on Hybrid Photonic/X Neurointerfaces in *Neurophotonics* Volume 9 Issue 3.

## 2 Diverse Imaging Modalities for Investigating Brain Function

In this section, we summarize a number of different imaging approaches for studying the organization and function of the brain. We will highlight the strengths and weaknesses of each [e.g., spatiotemporal resolution, brain coverage, and field of view (FOV)], emphasizing those of particular relevance to multimodal implementations.

### 2.1 Fluorescence Imaging

A number of optical imaging methods have been applied to the study of dynamic brain function, including fluorescence, luminescence, and intrinsic signal modalities.^10^^,^^11^ In this review, we limit our discussion to fluorescence imaging, given the recent advances in combining this approach with other methods. Fluorescence refers to the emission of photons by molecules that have previously absorbed light. Many fluorescent molecules alter their sensitivity or spectral range as a function of their atomic conformation, a phenomenon exploited in the development of reporters for neuronal activity.^11^^–^^14^ Given the electrical nature of neural signaling, fluorescent reporters of membrane voltage were prominent in early imaging studies that revealed patterned activity associated with spontaneous and sensory-driven network dynamics.^15^^–^^18^ However, higher signal-to-noise ratio (SNR) properties have led to the greater adoption of molecules that shift their fluorescent properties upon binding ionic calcium (${\mathrm{Ca}}^{2+}$). Neuronal depolarization can drive the opening of voltage gated ${\mathrm{Ca}}^{2+}$ channels, leading to increased fluorescence of cytosolic indicator molecules. Indeed, the large SNR afforded by many ${\mathrm{Ca}}^{2+}$ indicators has made them a critical, albeit indirect sensor of activity at the cellular and network scale.^12^^,^^19^^,^^20^ Given the key role of ${\mathrm{Ca}}^{2+}$ indicators in many recent studies using multiphoton and mesoscopic imaging, we will primarily focus on this class of probes.

A standard challenge to the use of fluorescent ${\mathrm{Ca}}^{2+}$ indicators is the need to introduce them to the intracellular compartments of single neurons. Early work in this field relied on loading neurons with organic indicators (e.g., Fura, Fluo, or Oregon Green-BAPTA dyes) through either a micropipette or extracellular application of lipophilic variants that could cross plasma membranes. More recently, the advent of genetically encoded indicators (e.g., GCaMP) has enabled the use of viral vectors or transgenic animals to target expression of fluorescent probes to specific brain regions and cell types.^11^^,^^12^ Additionally, the development of spectrally segregated indicators has opened the possibility of multicolor imaging in the same preparation.^20^

#### 2.1.1 Wide-field “mesoscopic” imaging

Fluorescent ${\mathrm{Ca}}^{2+}$ imaging has been used to measure neuronal activity at a variety of spatial scales, ranging from single synapses, to local populations of individual neurons, to large-scale neuronal networks spanning multiple brain areas. Mesoscopic imaging, a wide-field approach, has seen recent and rapid growth due to its relative ease of application in behaving animals.^21^ Fundamentally, mesoscopic imaging offers a compromise between spatiotemporal resolution and FOV. Fluorescence is collected from the brain surface, either through a cranial window or intact skull, and image formation occurs via a microscope-coupled camera. A typical FOV can span most of the mouse neocortex with single pixels corresponding to the spatially averaged activity of neurons located over a few tens of microns and an acquisition rate of 10 to 50 frames per second. The relatively low cost of necessary hardware (essentially a low-magnification objective and high-sensitivity camera) is a major advantage of mesoscopic versus other modalities, such as multiphoton imaging (Sec. 2.1.2).

Limitations of mesoscopic imaging largely stem from the modest resolution (driven by both camera pixel depth and light scattering in brain and skull tissue) that prevents analysis of single-cell activity. By comparison, the spatial resolutions of local-field potentials and functional MRI (fMRI) (Sec. 2.2) are several hundred microns or more,^22^^,^^23^ suggesting that mesoscopic ${\mathrm{Ca}}^{2+}$ signals are a robust option for reporting local circuit dynamics. Posing another challenge, fluorescence signals collected from the cortical surface are biased to more superficial cells given the strong scattering of photons in brain tissue limiting collection form deeper areas. In addition, signals represent a mix of emission from subcellular compartments, originating from somatic, dendritic, and axonal compartments, potentially impacting data interpretation. However, the ongoing development of indicators genetically targeted to specific cortical layers or subcellular compartments will likely drive substantial improvements in this approach.^24^^,^^25^ Finally, mesoscopic fluorescence signals are potentially contaminated by activity-dependent changes in light absorption due to fluctuating blood oxygenation.^26^ However, a number of strategies for minimizing the hemodynamic impact have been developed, and the overall degree of contamination remains poorly quantified.^21^^,^^26^^,^^27^

#### 2.1.2 Multiphoton imaging

Wide-field imaging relies on conventional fluorescence, where a single photon is absorbed then emitted by an electron. In multiphoton excitation, two or more photons are absorbed, resulting in subsequent emission. The nonlinear probability of multiphoton absorption limits the functional excitation volume in the sample, enhancing the axial imaging resolution, particularly in scattering tissue.^28^ The high (<1  μm) resolution of multiphoton fluorescence imaging enables monitoring activity from single neurons and subcellular compartments. Moreover, excitation is carried out via point scanning, and the “descanned” emitted light is collected via photomultiplier tubes, with image formation being done *post hoc*, significantly increasing the efficiency of light collection. The combination of this high resolution with genetic targeting of specific cell types, along with the ability to carry out studies in awake, head-fixed animals, has made multiphoton ${\mathrm{Ca}}^{2+}$ imaging a dominant workhorse technique for relating neuronal activity to behavior.

Limitations of multiphoton imaging include the high cost of equipment, which includes a high-power pulsed laser source for excitation. In addition, the high imaging resolution is only tenable after a craniotomy (removal of the optically scattering skull). Indeed, multiphoton imaging is limited to a depth of several hundred microns, necessitating tissue removal or insertion of an invasive lens to gain access to deeper brain structures.^28^^–^^30^ Recent developments in three-photon imaging have extended the accessible depths, but availability of fluorophores and laser sources compatible with this modality remain limited.^31^ Finally, the FOV accessible with point-scanning is limited, with most multiphoton systems providing $<1\;{\mathrm{mm}}^{2}$. Nevertheless, newer strategies for increasing the field of view while maintaining cellular resolution are in constant development, though typically with the challenge of complex optical designs.^32^^,^^33^

#### 2.1.3 Fiber photometry

Fiber photometry is a variant of fluorescence signal collection that does not involve explicit image formation.^34^ Instead, a fiber optic is implanted within the brain, allowing the delivery of excitation light and the collection of emitted photons as a bulk signal from all fluorescent cells near the fiber end. The power of this approach is the ability to target deep brain regions not accessible to surface imaging with modest invasiveness (typical fiber diameters are ∼100 to 200  μm). In addition, the placement of multiple fibers allows simultaneous signal collection from spatially distinct targets.^35^ As there is no scanning or camera required, temporal resolution is essentially limited only by indicator kinetics, and this approach can also take advantage of genetically encoded probes to limit signals to specified cell types. Nevertheless, the lack of image formation precludes strong inferences about the cellular and subcellular nature of the emitted signals.

### 2.2 Magnetic Resonance Imaging

This mode leverages the phenomenon of nuclear magnetic resonance (MR)—the absorption and emission of electromagnetic waves at characteristic frequencies in the radio frequency (RF) range by nuclei within a strong magnetic field. Both humans and animals are routinely examined by MR imaging studies making this mode useful for interspecies translation. MR imaging can capture diverse structural and functional brain features with full organ coverage noninvasively. As an established neuroimaging technology, there are many comprehensive reviews on a wide variety of specialized topics. The spatiotemporal resolution of MR images depends on the source of contrast, hardware, and (as with optical imaging) the priorities of the acquisition (e.g., spatial resolution can be sacrificed for temporal resolution). As a benchmark, the spatial and temporal resolution limits of MR data are more coarse than optical imaging data. Further, although the sources of MR contrast are many, they are fewer and often less specific than those available from optical modes. Here we focus on one source of contrast: the blood-oxygen-level-dependent (BOLD) signal as it is a dynamic measure of brain activity and therefore well-suited to being simultaneously acquired with the other modalities discussed in this review.

BOLD is a nonspecific, or summary, metric of brain activity, which arises from local changes in blood oxygenation. The imaging sequences used to collect these data are sensitive to the effect paramagnetic deoxyhemoglobin (Hb) has on local water. The BOLD signal is inherently slow due to the underlying biology of the hemodynamic response to local activity (adequately sampled at ∼1  Hz). The data has low SNR and spatial resolution (∼100 to $1000\;{\mu m}^{3}$) when compared to optical imaging modes. In a typical experiment, a voxel contains a few million neurons^36^ or roughly corresponds to the FOV of a multiphoton acquisition.^37^ For a more in-depth discussion of the BOLD signal, the interested reader is referred to Buxton 2009.^38^

Despite being an established neuroimaging technique, BOLD-fMRI is a highly active field with researchers working to improve the raw data—through faster acquisitions,^39^ higher spatial resolution,^40^ and ways of reducing artifacts (e.g., susceptibility-caused distortions^41^). In parallel, ways of conducting experiments in animals that more closely mimic human neuroimaging (e.g., eliminating the use of anesthesia to reduce subject motion^42^), and leveraging complementary simultaneous techniques (as discussed here) are at the cutting edge of BOLD-fMRI basic science applications.

## 3 Multimodal Implementations

As discussed above, each of the various approaches for imaging neural activity has distinct advantages and disadvantages, largely in the realm of spatiotemporal resolution, cell-type specificity, and the relationship between the signal and underlying neural activity. However, recent efforts to combine different methods in the same experimental preparation have opened new avenues for investigating the nervous system, attempting to synergize the strengths of each component. Now, we summarize these efforts to highlight the benefits of multimodal strategies, focusing on combinations of mesoscopic imaging with either multiphoton imaging or fMRI.

### 3.1 Simultaneous Mesoscopic and Multiphoton Imaging

**Fig. 1:**
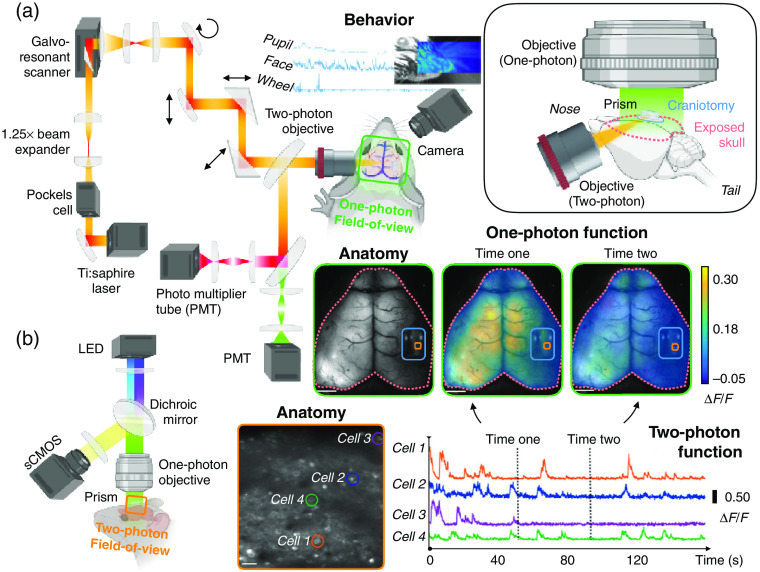
Simultaneous one-photon (wide-field) imaging and two-photon imaging. (a) A schematic of the light path of the two-photon imaging microscope (left). The FOV of the one-photon imaging setup is indicated by a green box. Behavioral data are collected using an auxiliary camera (middle). A schematic of the surgery—skull thinning, cranial window, and placement of a small prism—is shown in the in-lay (upper right). (b) A schematic of the light path of the one-photon imaging microscope (left). The FOV of the multiphoton imaging setup is indicated by the orange box. Example data are shown (right) (adapted with permission from Refs. 43 and 44).

Mesoscopic fluorescence imaging provides a high temporal resolution readout of activity across the entire dorsal surface of the mouse cortex but nevertheless lacks spatial resolution sufficient to report single cell dynamics. Yet, a critical goal in neuroscience research is to understand how the activity of individual neurons emerges within the larger network context, requiring an experimental window into both spatial scales. To this end, we recently developed an approach for fluorescent monitoring of neural activity across several orders of magnitude of spatial scale by combining mesoscopic and multiphoton ${\mathrm{Ca}}^{2+}$ imaging of genetically encoded indicators in the mouse neocortex (Fig. 1).^43^ Wide-field imaging was performed with a standard upright system, whereas multiphoton imaging was carried out by implanting a small microprism on the brain surface, allowing us to direct the two light paths orthogonally to each other. With this approach, we monitored the firing of single neurons in the somatosensory cortex while observing the large-scale network dynamics of the entire cortical surface. Results showed great heterogeneity in the functional connectivity of neighboring neurons with specific cortical networks that also varied between genetically targeted excitatory and inhibitory cells. The connectivity of cells with the larger cortical architecture was also modulated by behavioral state, echoing findings from work combining wide-field imaging with electrophysiology.^45^^–^^47^ A major focus of neuroscience has been the classification of single neurons by their molecular, anatomical, and functional properties, such as tuning of activity to behavior or sensory inputs.^48^^–^^50^ This combined imaging approach now allows us to further characterize cells by their connectivity to both local and large-scale networks.

### 3.2 Combining Various Modes with fMRI

A strong motivation for combining fMRI with complementary simultaneous approaches has been to gain a better biological understanding of the BOLD signal. The BOLD signal is beholden to the cellular and vascular constituents of the tissue—herein, the neurogliovascular unit (NGVU). To what extent BOLD signals are driven by any individual component of the NGVU is unresolved and may depend on brain region,^51^^,^^52^ type of activity (spontaneous or stimulated),^53^ developmental stage,^54^ and the vascular as well as cellular health of the tissue.^55^ This is a mixed blessing. On one hand, determining which components are noise and which are signal is challenging,^56^ as it is inferring the biological drivers of BOLD signal changes. On the other hand, the BOLD signal is a rich whole brain measure of overall function that is sensitive to various aspects of tissue health.

The potential impact of a better understanding of the BOLD signal is far-reaching given that this measure offers noninvasive whole-organ coverage and is widely used across species, thus providing a link between basic research and patient management. Further, there is significant evidence, although much of it is correlative, that brain network measures derived from BOLD contrast (i.e., functional connectivity) may have utility as a diagnostic and prognostic tool for a variety of neurological conditions as well as brain injuries and diseases (see select reviews by Wang et al.,^57^ Lunkova et al.,^58^ Harikumar et al.,^59^ Yoon et al.,^60^ and Kanel et al.^61^). The combination of fMRI with complementary modes not only helps discern what measures may be most useful clinically but also stands to yield mechanistic insight into the biology that underpins BOLD signal differences between individuals, populations, and time points.

A decade before the advent of combining fMRI with optical modes (Secs. 3.2.1 and 3.2.2), concomitant electrophysiology, or electroencephalography (EEG) was being implemented to obtain simultaneous measures of neural activity and BOLD signals.^4^^,^^56^^,^^62^^–^^64^ Electrophysiology and EEG measure current or voltage changes caused by the movement of charged ions within biological samples. Critically, these modes are sensitive to action potentials, as well as concert activity, but they do not offer cell-type specificity.^65^ Although electrophysiology and EEG persist as a choice means of measuring the neural contribution to the BOLD signal in humans (due to the invasiveness of fluorescent probes), optical approaches are a quickly evolving and powerful alternative for basic research applications. Due to the plethora of targetable fluorescent probes, optical modes can interrogate the contributions of specific neural subpopulations (e.g., excitatory or inhibitory interneurons), as well as other cellular (e.g., glial), vascular, and molecular contributors to the BOLD signal.^66^ fMRI and simultaneous fiber-photometry or wide-field optical imaging are discussed below; as to the best of our knowledge, multiphoton imaging and fMRI have only been described in theory.^67^

#### 3.2.1 Photometry and BOLD fMRI

The combination of these modalities enables signal from the whole brain (via fMRI) to be collected with a point measurement (via fiber photometry) of cell-type specific activity and/or the ability to modulate activity within the small target region. These experiments enable the role a small target region to be interrogated in the context of whole brain network activity.

Simultaneous fiber photometry and MRI are relatively straight forward and cost-effective to implement. The cell-type specificity, developing arsenal of fluorescent tools, and relative compatibility with MRI (compact and metal-free) make fiber-photometry a useful mode for probing regional activity with concomitant MR measures. The principal limitations of fiber-photometry are poor coverage that this mode is not image forming and invasiveness. Broadly, the application of simultaneous fiber-photometry and fMRI takes two forms: (1) termed “optogenetic-fMRI,” where the activity of a target cell population and/or region is sensitized to a specific wavelength of light to enable activity modulation^68^ and (2) passive measurement of activity (either spontaneous or elicited through exogenous stimulation, e.g., electrical stimulation of the fore/hind limbs).

Optogenetic-fMRI (1), described for the first time in 2010 by Lee et al.,^69^ has been used to probe the BOLD signal through the actuation of activity within target regions and to interrogate long-range connectivity (see recent reviews by Lee et al.,^70^ Albers et al.,^71^ and Snyder and Bauer^72^). Notably, this approach was used to demonstrate that the BOLD signal has both neural as well as glial contributors.^69^^,^^73^^–^^76^ Experiments that monitor rather than drive activity, (2), are a bit more recent (see select publications by Tong et al.,^53^ Schulz et al.,^73^ Schmid et al.,^74^ Liang et al.,^75^ Wang et al.,^76^ Schwalm et al.,^77^ Schlegel et al.,^78^ and Ma et al.,^79^ and the review by Wang et al.^80^). These studies include quantifications of task versus rest related differences in multimodal signal correspondence^53^ and noise versus signal components in the fMRI global signal.^53^^,^^79^

#### 3.2.2 Wide-Field and Simultaneous fMRI

Combining whole brain fMRI and one-photon optical imaging enables widespread circuit and network activity to be interrogated using different complementary sources of contrast. As discussed above, the BOLD signal is a cell-type agnostic measure of brain activity; whereas targeted fluorescence offers the ability to interrogate the activities of specific cell populations. Gaining a better understanding of the neurobiological contributors to the BOLD signal has far-reaching implications for fMRI research and clinical use cases. Conversely, wide-field optical imaging modes cannot access deep brain structures that are contained within the fMRI FOV.

Two groups have described implementations of simultaneous single-photon optical imaging and fMRI (see Table 1).^81^^–^^83^ Together, these works show that these modes can access a variety of optical and fMRI contrasts, where data can be collected from different species, using different MR scanner strengths, as well as an acute or longitudinal study design. Both groups utilize anesthesia, custom RF hardware, a fiber bundle to relay optical images out of the scanner, and skull thinning to improve optical signal transmission. These points of methodological convergence indicate common solutions to challenges that are inherent to the combination of these modes. Postprocessing approaches followed by both groups used steps optimized for unimodal studies. Vascular landmarks are used for cross-modal data registration, and a generalized linear model is used to identify pixels and voxels that respond to exogenous stimulation. Modeling approaches implemented by both groups take optical data as input and output a prediction of the BOLD signal that is compared to the measurement. Each study finds good cross-modal correspondence in responding region topography and time-course prediction.

**Table 1:** Studies that implement simultaneous wide-field and fMR imaging.

	Kennerly et al. 2005	Kennerly et al. 2012	Lake et al. 2020
Species	Rats	Rats	Mice
Anesthesia	Urethane	Urethane	Isoflurane
Paradigm	Acute	Acute	Acute and chronic
Stimulus	Whisker and ${\mathrm{CO}}_{2}$	Whisker	Hind-paw
Surgery	Skull thinning	Skull thinning	Skull thinning
Optical			
Spatial resolution ($\mu m^{2}$)	200 × 200	80 × 80	25 × 25
Temporal resolution (ms)	125	125	100
Coverage	6-mm diameter circle	6-mm diameter circle	$14.5\times 14.5\;{\mathrm{mm}}^{2}$
Contrast	Intrinsic oxy-/deoxy-Hb	Intrinsic oxy-/deoxy-Hb	Fluorescence ${\mathrm{Ca}}^{2+}$
Fiber bundle	50,000	50,000	2,000,000
fMRI			
Spatial resolution	$0.47\times 0.47\times 2.00\;{\mathrm{mm}}^{3}$	$0.47\times 0.47\times 2.00\;{\mathrm{mm}}^{3}$	$0.4\times 0.4\times 0.4\;{\mathrm{mm}}^{3}$
Temporal resolution (s)	1 or 2	1	1
Coverage	Single slice	Single slice	28 slices
Contrast	BOLD and CBV	BOLD	BOLD
Scanner (T)	3	7	11.7
RF-coil	Custom	Custom	Custom

**Fig. 2:**
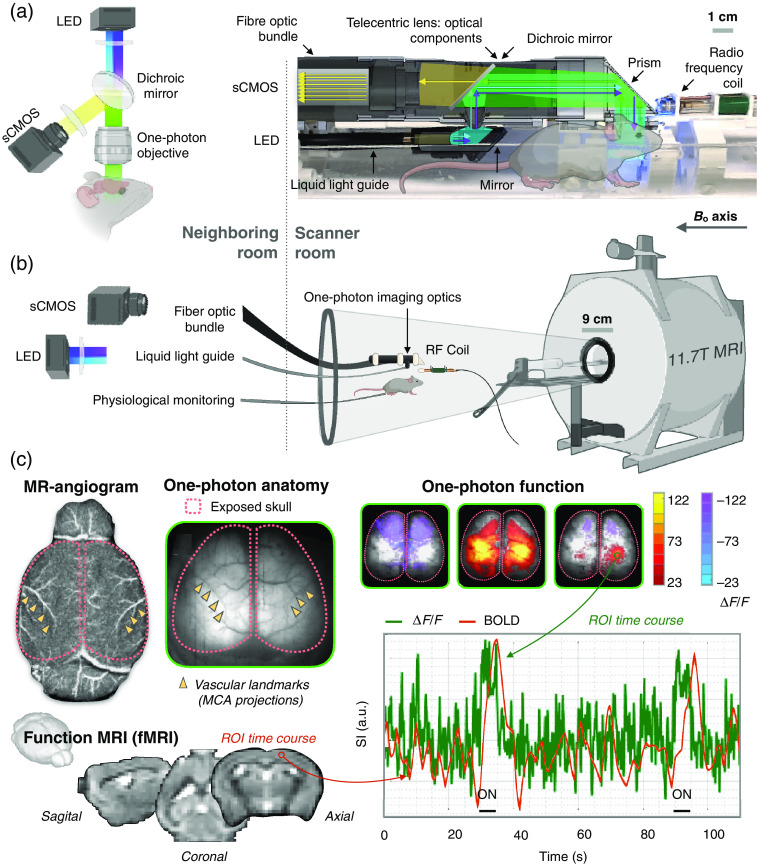
Simultaneous one-photon optical imaging and fMRI. (a) A schematic for unimodal one-photon optical imaging (left) and an adaptation for acquiring these data within the MR scanner (right). (b) An overview of the multimodal setup. (c) Example anatomical images for multimodal registration and functional data from unilateral hind-paw stimulation (adapted with permission from Ref. 83).

There are also unique contributions from each study. The works by Kennerly et al.^81^^,^^82^ focus on stimuli elicited responses and the correspondence between intrinsic oxy-/deoxy-Hb optical signals and BOLD or cerebral blood volume (CBV) contrast. A focus of these works is to quantify the vascular component of the BOLD signal. In both studies by Kennerly et al.,^81^^,^^82^ responses to stimuli are averaged, and most findings are averaged across subjects (response topographies being the one exception^82^). Lake et al.^83^ measured fluorescent ${\mathrm{Ca}}^{2+}$ signal from excitatory neurons—a different component of the NGVU that is further removed from the BOLD signal than oxy-/deoxy-Hb. This work reports averaged responses to stimuli, but also a quantification of individual responses (no temporal averaging), and measures of spontaneous activity. With the large FOV in Lake et al.’s^83^ study, this includes analyses of cortical network architecture. Together, these complementary studies establish a foundation for this multimodal approach (Fig. 2).

#### 3.2.3 Future work for multimodal imaging approaches

The combination of optical contrasts (e.g., intrinsic oxy-/deoxy-Hb and fluorescent imaging) to capture vascular and cellular contributors in one acquisition optically with concomitant BOLD or CBV contrast is a logical next step for simultaneous wide-field and MR imaging. Further, ${\mathrm{Ca}}^{2+}$ imaging in excitatory neurons^81^ is one of many available fluorescent indicators. Implementing the same acquisition methods described by Lake et al.,^83^ it is feasible to tackle a wide array of neuroscience questions using different indicators as well as the combination of multiple indicators—targeting different cell-types (e.g., excitatory neurons as well as inhibitory interneurons, and glia), sources of contrast (e.g., ${\mathrm{Ca}}^{2+}$ or voltage), or brain regions (e.g., different cortical layers). These data will help deepen our understanding of NGVU contributors to cortical fMRI signals. Interrogating the relationship between cortical signals and deeper brain structures is another area where the co-application of optical modes and fMRI stands to make a contribution in the near future. There are also many opportunities for advances in how to postprocess and analyze these multimodal data.

Applying simultaneous single-photon and MR imaging to measure development, age, injury, disease, or treatment related changes in brain networks are also clear avenues for high-impact future discoveries given the specificity of fluorescent indicators and the cross-species translatability of fMRI contrasts. To these ends, expanding the application of these multimodal approaches to awake animals will be a key near future methodological development. Experiments in awake animals remove the confounding effects of anesthesia (see reviews by Lecoq et al.^84^ and Gao et al.^42^) and affords the ability to study brain activity during task performance.^85^ The longitudinal preparation in the Lake et al.^83^ study is compatible with the implementation of an acclimation protocol, which has been shown to reduce motion and stress in murine fMRI experiments.^37^^,^^85^^–^^94^ Toward these objectives, there has been some recent pioneering work in both unimodal (murine fMRI)^37^^,^^85^ and multimodal (fiber photometry and fMRI)^95^ experimentation.

**Fig. 3:**
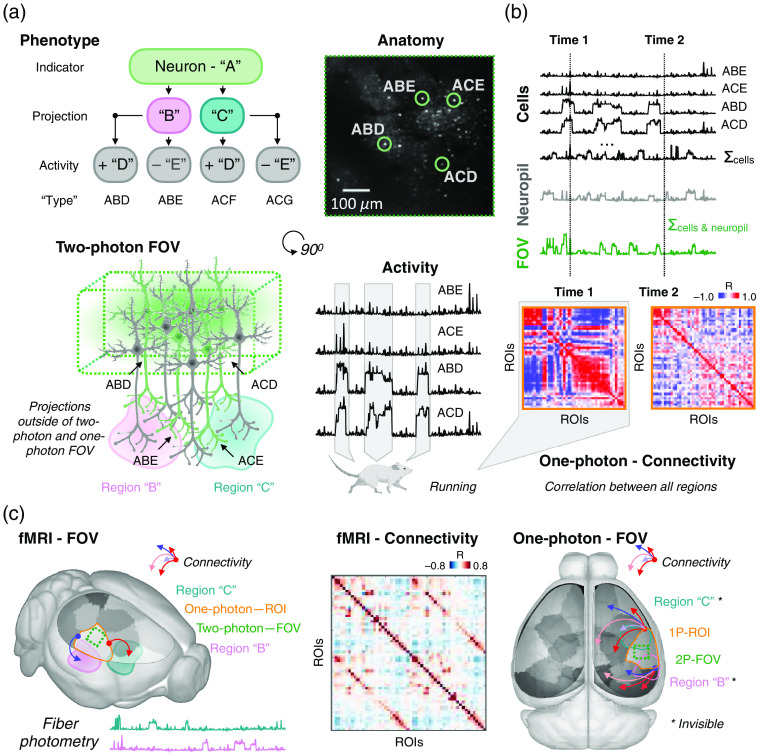
Linking across spatiotemporal scales—from (sub)cellular to the whole-brain—and ultimately to behavior. (a) Each neuron can be classified based on a variety of attributes (top left). In this toy example, neurons are labeled with a fluorescent indicator “A.” To further define neurons of “type-A,” they can be grouped by whether they project to two remote brain regions: “B” and “C.” Further, neurons of type-A (that project to region B or C) may (+) or may not (−) exhibit modulated activity during animal locomotion. Together, these three attributes (fluorescence, projection, and modulation with locomotion) can define the phenotype of a cell. To access these attributes can require more than one imaging mode (bottom left) and an understanding of how behavior modulates activity (bottom right). In our example, regions B and C may be deeper in the brain than one- or two-photon imaging can access. The activity of individual cells within a typical multiphoton microscope FOV (top right) sums to approximate coarser measures (b). Understanding how to translate short- and long-range circuit and network function from cellular to mesoscale concert activity, as well as how these measures (e.g., functional connectivity) reflect behavior, will come with help from simultaneous multimodal implementations. (b) Single-cell measures (top) in a two-photon FOV can be summed to approximate measures accessible by one-photon imaging (bottom). Relationships between cells (two-photon) or regions (one-photon) can be summarized using a correlation (connectivity) matrix; a measure of activity synchrony (or asynchrony) between all region pairs. Connectivity matrices can be computed from different epochs of an experiment. In our example, epochs of locomotion or rest using data from different modes. Extending this understanding to the whole brain (c) will also require the implementation of complementary optical approaches and fMRI. For access to deep brain regions (like B and C), either fMRI or fiber photometry can be implemented; the relative coverage and FOV of the imaging technologies discussed here are summarized in (c) side (left) and top (right) view. Like optical imaging data, fMRI data can be summarized using a connectivity matrix (middle), which can help to translate complementary information across spatiotemporal scales.

The utility of multimodal approaches based on fluorescence imaging is likely to benefit significantly from ongoing technical innovations. For example, the generation of innovative reporters for neuronal activity, namely genetically encoded reporters for fast transmitters, such as glutamate and GABA,^96^^–^^98^ and neuromodulators, such as norepinephrine, acetylcholine, and dopamine,^99^^–^^102^ will expand our ability to link cellular dynamics with behavior. Additionally, the continued generation of transgenic mouse lines based on the targeted expression of CRE recombinase^103^^,^^104^ will enable imaging of distinct cortical cell types, e.g., simultaneously monitoring large-scale network organization of excitatory and inhibitory cells. Similarly, innovative viral strategies to label cells based on projection targets^105^^,^^106^ or cell type-specific promoters^107^^,^^108^ will further expand the power of both wide-field and cellular-resolution imaging strategies. Finally, these advances in fluorescence imaging also hold great promise for their coimplementation with fMRI. Through an increasingly more detailed understanding of subcellular through whole-brain circuit and network-level function, we stand to improve our collective ability to link behavior to clinically accessible measures (fMRI) and the underlying, pharmacologically actionable, drivers (Fig. 3).

## 4 Discussion

To implement imaging modes simultaneously, technical, physical, and analytical obstacles must be overcome. The optical imaging modes offer access to an impressive array of structural and functional contrasts with high SNR and specificity, whereas their combination with fMRI offers a link to human neuroimaging as well as whole-organ coverage. Although optical imaging approaches operate within a bounded spatiotemporal milieu, with less than whole-organ coverage, advances in the field are constantly extending the imaging capabilities of these modes. Multimodal optical implementations offer a unique ability to probe (and manipulate) the circuits of which global brain organization is composed. With access to cellular (and subcellular) resolution as well as mesoscale activity, optical modes can bridge across an impressive swath of the spatiotemporal spectrum. Further, optical imaging experiments are routinely performed in awake (sometimes freely exploring) subjects allowing for simultaneous measures of physiological state and behavior (see review by Lecoq et al.^84^). However, the invasiveness of these modes limits their application to animal models, which is one of the strongest motivations for combining these approaches with those accessible to human subjects. MRI is routinely implemented in animal as well as human studies making it useful for interspecies translation. To this end, the studies that implement simultaneous fiber photometry or single-photon imaging and MRI have taken the first steps in gaining deeper insight into the underlying cellular and mesoscale activity, which supports widespread global brain function and underpins fMRI contrasts. We anticipate substantial growth and development in the implementation of simultaneous multimodal methods in the near future.

To this end, it has long been appreciated that there are both quantitative and qualitative differences between anesthetized and waking conditions.^109^^,^^110^ Moreover, waking itself has been divided into quiet and active periods.^111^^–^^115^ Active waking (arousal) has been operationally characterized by epochs of movement (e.g., wheel running) and pupillary dilation, providing easily accessible experimental parameters.^110^ Arousal corresponds to neuronal membrane depolarization, multiplicative increases in the gain of sensory-responsiveness, and enhanced SNRs.^110^ Recent work using mesoscopic imaging has also revealed state-dependent fluctuations in cholinergic signaling across the cortex and corresponding reorganization of functional connectivity in cortical networks,^44^ whereas simultaneous mesoscopic and two-photon imaging has shown state-dependent reorganization of correlations between single cells and large-scale networks.^43^ Continued parallel development of methodologies monitoring and analyzing neural activity and behavior is likely to further expand our understanding of the links between these phenomena.

A concerted shift by optical imaging labs toward imaging awake and behaving subjects has been paralleled by sparse progress to reduce the use of anesthesia during fMRI experiments. Apart from the pioneering work from a handful of labs,^37^^,^^85^^–^^94^ most fMRI data are still acquired under anesthesia to control for subject motion and stress. Due to the exceptional noise produced by fMRI (>120  dB) and the necessity to keep the animal immobile to obtain high-quality data, extensive acclimation protocols have proven necessary; but a consensus on the best practices and quality standards has yet to be reached. Nevertheless, given the established effects of anesthesia on neurophysiology and neurovascular coupling, the fact that complementary modalities (optical imaging) are opting for awake protocols, and that human subjects are rarely imaged under anesthesia, there are considerable gains to be made from conducting fMRI experiments (and multimodal implementations) in awake subjects. We expect that labs implementing multimodal approaches, which combine optical and fMRI data acquisition, will move toward implementations in awake subjects in the near future.^94^ Another encouraging future direction along which we anticipate growth is the use of multimodal imaging strategies to add to our collective understanding of the cellular contributors to the BOLD-fMRI signal.^83^^,^^116^^–^^118^ Specifically, the use of multiple fluorophores (to label different cells) in combination with fMRI has the unique ability to disentangle the different cellular populations, which contribute to the BOLD signal.
